# Outcomes of Double-Hit and Single High-Risk Cytogenetic Newly Diagnosed Myeloma in Transplant-Eligible Patients

**DOI:** 10.3390/curroncol33050246

**Published:** 2026-04-26

**Authors:** Raeef Rahman, Anas Zayad, Carmel Awadallah, Beining Wang, Jianzheng Wu, Aroog Khaliq, Prerna Mewawalla, Shebli Atrash, Dinesh Pal Mudaranthakam, Joseph McGuirk, Zahra Mahmoudjafari, Muhammad Umair Mushtaq, Al-Ola Abdallah, Nausheen Ahmed, Jordan Snyder, Anita Mazloom, Omar Alkharabsheh, Mansi R. Shah

**Affiliations:** 1 School of Medicine, University of Kansas, Kansas City, KS 66160, USA; rrahman@kumc.edu; 2 Department of Internal Medicine, Hamad Medical Corporation, Doha P.O. Box 3050, Qatar; 3 Division of Internal Medicine, St John Episcopal Hospital, Far Rockaway, NY 11691, USA; 4 School of Medicine at Shanghai Medical College, Fudan University, Shanghai 201399, China; 5 Division of Biostatistics and Data Science, University of Kansas Medical Center, Kansas City, KS 66205, USA; 6 Division of Hematologic Malignancies & Cellular Therapeutics, University of Kansas Medical Center, Westwood, KS 66205, USA; 7 Division of Hematology and Cellular Therapy, Allegheny Health Network Cancer Institute, Pittsburgh, PA 15224, USA; 8 Levine Cancer Center, Atrium Health, Wake Forest University School of Medicine, Charlotte, NC 28204, USA; 9 Division of Hematology and Oncology, Mitchell Cancer Institute, University of South Alabama, Mobile, AL 36604, USA; 10 Division of Hematology and Oncology, University of Cincinnati, Cincinnati, OH 45267, USA; 11 Division of Hematology, Rutgers Cancer Institute of New Jersey, New Brunswick, NJ 08901, USA

**Keywords:** high-risk multiple myeloma, double-hit cytogenetics, autologous stem cell transplantation, survival outcomes

## Abstract

Introduction: High-risk multiple myeloma (HRMM) is primarily defined by adverse cytogenetic abnormalities that are associated with inferior outcomes despite contemporary treatment with novel induction agents and autologous stem cell transplantation (ASCT). While the accumulation of high-risk lesions has been proposed to further stratify prognosis, the relative impact of single-hit versus double-hit HRMM on post-transplant outcomes in real-world clinical practice remains incompletely characterized. Methods: We conducted a multicenter retrospective cohort study of transplant-eligible adults with HRMM undergoing upfront ASCT between 2009 and 2024 at three U.S. academic centers. Patients were categorized as single-hit HRMM or double-hit HRMM based on the number of high-risk cytogenetic abnormalities at diagnosis. Response rates, progression-free survival (PFS), and overall survival (OS) were compared between groups using standard statistical methods. Results: A total of 154 patients were included, of whom 63 had SH-HRMM, and 91 had DH-HRMM. Following induction therapy, overall response rates (ORR) were high and comparable between groups. After ASCT, ORR was significantly higher in SH-HRMM compared with DH-HRMM (97% vs. 82%, *p* = 0.006), although depth of response did not differ significantly. With a median follow-up of 150 months, median OS was 103 months in SH-HRMM and 94 months in DH-HRMM (*p* = 0.40). Median PFS was 36 months and 31 months, respectively (*p* = 0.20). Restricted mean survival time analyses similarly demonstrated no significant differences in OS or PFS between groups. Although ORR differed, depth of response and long-term survival did not. Conclusions: In this real-world cohort of transplant-treated HRMM, single-hit disease was associated with a higher likelihood of achieving post-ASCT response; however, no significant differences in long-term survival outcomes were observed between single-hit and double-hit HRMM. These findings suggest that cytogenetic hit burden alone may be insufficient to predict post-transplant survival, highlighting the need for refined risk stratification incorporating additional biological and response-based markers.

## Simple Summary

Multiple myeloma is a blood cancer where certain genetic abnormalities carry a worse prognosis. Previous studies have suggested that patients with two or more genetic abnormalities had significantly worse outcomes than those with only one. In this retrospective cohort of 154 high-risk patients who underwent stem cell transplantation, we compared these groups in a modern clinical setting. Unexpectedly, the research found no significant difference in survival or disease control between patients with one high-risk genetic abnormality and those with two or more. These findings suggest that contemporary treatments, including newer drug combinations and ongoing therapy after transplant, may be narrowing the survival gap for patients with more complex genetic risks. It also suggests that simply counting genetic abnormalities may be insufficient for predicting outcomes today. This work encourages future studies to develop more precise tools to guide personalized treatment for these challenging cases.

## 1. Introduction

Multiple Myeloma (MM) is a malignant plasma cell dyscrasia characterized by uncontrolled proliferation of monoclonal plasma cells in the bone marrow. Though long-term outcomes of patients with MM have improved significantly due to the development of various therapies, most patients ultimately go on to relapse [1]. For transplant-eligible adults, contemporary intensive therapy commonly incorporates multi-agent induction followed by autologous stem cell transplantation (ASCT), with or without consolidation, and then maintenance, a sequence that has contributed to prolonged survival in the modern era [1]. Therapy in this cohort reflects PI-based induction, routine maintenance, and access to anti-CD38 and T-cell-directed salvage therapies. Despite these therapeutic advances, a substantial subset of patients experience early relapse and premature death, underscoring the continued prognostic importance of cytogenetic and genomic risk stratification [1].

High-risk multiple myeloma (HRMM) is mainly defined by adverse cytogenetic abnormalities. However, other clinical and biological features have also been incorporated into risk-stratification tools such as the Mayo Clinic mSMART model, which account for extramedullary disease and markers of aggressive disease biology [1]. In recent years, various risk stratification tools have been developed to categorize patients with MM by disease features and to more accurately evaluate prognosis. These developments have resulted in improved patient categorization by disease feature and subsequently more accurate prognoses.

Risk classification scores identify specific cytogenetic abnormalities as high-risk, including deletion 17p [del(17p)], immunoglobulin heavy chain translocations such as t(4;14), t(14;16), and t(14;20), and chromosome 1 abnormalities (which includes gain/amplification of 1q, deletion of 1p [del(1p)]), with additional adverse impact from TP53 abnormalities and high clonal burden of del(17p) [2]. Recent evidence demonstrates that the co-occurrence of high-risk lesions can amplify risk beyond any single abnormality, supporting an “accumulation of hits” model of aggressive disease pathogenesis [2,3]. Building on this concept, clinical definitions frequently categorize “double-hit” myeloma (DHM) as the presence of any two high-risk factors and “triple-hit” disease as three or more, although the precise composition of “high-risk factors” and technical thresholds vary across studies [4,5,6].

Prior studies have consistently demonstrated inferior PFS and OS in patients with DHM compared with those with single-hit or standard-risk disease, including in cohorts treated with frontline ASCT and post-transplant maintenance [2,6,7]. However, data specifically evaluating the prognostic impact of double-hit cytogenetics in transplant-treated high-risk populations in the contemporary maintenance era remain limited. We therefore conducted a multicenter retrospective cohort study of transplant-eligible adults with high-risk multiple myeloma undergoing upfront autologous stem cell transplantation to compare post-transplant outcomes between patients with single-hit and double-hit high-risk cytogenetics and to characterize response and survival in a contemporary real-world population.

## 2. Methods

### 2.1. Study Design and Setting

This was a retrospective, multicenter cohort study evaluating real-world outcomes of upfront autologous stem cell transplantation (ASCT) in patients with single-hit versus double-hit high-risk multiple myeloma (HRMM). Patients were identified from three U.S. academic medical centers: the University of Kansas Medical Center (KUMC), Allegheny General Hospital (AGH), and Atrium Health Levine Cancer Institute (LCI), in collaboration with the United States Myeloma Innovation Research Collaboration (USMIRC).

Patients who underwent upfront ASCT between January 2009 and January 2024 were identified using institutional electronic health records and transplant databases. The study was conducted in accordance with institutional review board approvals at all participating centers.

### 2.2. Patient Population

Adult patients (≥18 years) with MM who underwent upfront ASCT were eligible for inclusion. Diagnosis was confirmed according to International Myeloma Working Group (IMWG) criteria [8]. Eligible patients were required to have measurable disease at diagnosis, defined by quantifiable serum or urine monoclonal protein or an abnormal involved serum free light chain level with an abnormal ratio. Patients were considered transplant-eligible based on clinical criteria and baseline organ function requirements. Other inclusion criteria included: absolute neutrophil count, ≥1.0 × 109/L; hemoglobin level, >7.5 g/dL; platelet count, ≥75 × 109/L (≥50 × 109/L if ≥50% of the bone marrow was infiltrated with MM cells); alanine aminotransferase and aspartate aminotransferase levels <2.5 times the upper limit of normal; total bilirubin level, <1.5 times the upper limit of normal; creatinine clearance, ≥30 mL/min; and corrected serum calcium, ≤14.0 mg/dL (≤3.5 mmol/L).

Patients were excluded if they had received tandem or salvage ASCT, non-melphalan-based conditioning regimens, plasma cell leukemia (defined as circulating plasma cells ≥5% of peripheral blood leukocytes), central nervous system involvement, concurrent AL amyloidosis, another active malignancy within five years prior to diagnosis (with the exception of adequately treated localized non-melanoma skin cancer or in situ malignancies) uncontrolled active infection or significant comorbidity, receipt of prior systemic anti-myeloma therapy outside standard induction (excluding limited corticosteroids or localized radiotherapy), or missing baseline cytogenetic data required for high-risk classification.

#### 2.2.1. Cytogenetic Assessment and Risk Stratification

Cytogenetic risk assessment was performed on diagnostic bone marrow aspirate specimens obtained at initial diagnosis prior to initiation of therapy using conventional metaphase cytogenetics and interphase fluorescence in situ hybridization (FISH) according to institutional laboratory standards at each participating center. High-risk cytogenetic abnormalities included deletion 17p [del(17p)], translocations t(4;14), t(14;16), and t(14;20), gain or amplification of chromosome 1q, and deletion of chromosome 1p. FISH positivity thresholds followed institutional laboratory definitions, acknowledging variability in cutoff values across centers and over time. Cytogenetic risk classification was based on established definitions in use during the study period and at the time of data collection, recognizing that more recent IMWG/IMS consensus recommendations have further refined the definition of high-risk disease.

Patients were categorized based on the number of high-risk cytogenetic abnormalities present at diagnosis. Single-hit high-risk (HRMM) was defined as the presence of exactly one high-risk cytogenetic abnormality, whereas double-hit HRMM was defined as the presence of two or more high-risk cytogenetic abnormalities. Patients with three or more abnormalities were included within the double-hit group for analysis. Gain and amplification of chromosome 1q were analyzed as a single category because copy number thresholds distinguishing gain from amplification were not consistently reported across participating centers, particularly in earlier treatment eras.

#### 2.2.2. Treatment Exposure and Transplant Procedure

Patients received induction therapy according to institutional practice patterns prior to ASCT. All patients underwent conditioning with high-dose melphalan followed by ASCT. Post-transplant maintenance therapy was administered at the discretion of the treating physician and categorized by agent class. No patients in this cohort received consolidation therapy following induction or transplant; however, maintenance therapy was administered according to institutional practice. Treatment exposures were described to contextualize outcomes but were not used to define study groups.

#### 2.2.3. Outcomes and Response Assessments

The primary outcomes were progression-free survival (PFS) and overall survival (OS). PFS was defined as the time from ASCT to documented disease progression or death from any cause, whichever occurred first. OS was defined as the time from ASCT to death from any cause. Patients without an event were censored at the date of last follow-up.

Secondary outcomes included depth of response. Treatment responses and disease progression were assessed according to IMWG uniform response criteria.

### 2.3. Statistical Analysis

Descriptive statistics were used to summarize baseline demographic, clinical, and disease characteristics. Categorical variables were reported as frequencies and percentages, while continuous variables were summarized using medians with interquartile ranges (IQRs) or means with standard deviations (SDs), as appropriate. Comparisons between single-hit and double-hit groups were performed using Pearson’s chi-squared test or Fisher’s exact test for categorical variables and the Wilcoxon rank-sum test for continuous variables.

Survival outcomes were estimated using the Kaplan–Meier method and compared between groups using the log-rank test. All statistical tests were two-sided, with a *p* value < 0.05 considered statistically significant. Analyses were conducted using R software (The version is 4.3.1, R Foundation for Statistical Computing, Vienna, Austria).

### 2.4. Ethical Approval

This study was approved by the Institutional Review Board (IRB) at each respective institution. Informed consent was not considered given the retrospective review method used in the study. All data was de-identified to ensure patient confidentiality.

## 3. Results

### 3.1. Baseline Patient Characteristics

Baseline demographic, clinical, and disease characteristics of the study population are summarized in Table 1. A total of 154 patients were included in the analysis, comprising 91 (59%) patients with double-hit HRMM and 63 (41%) patients with single-hit high-risk disease. The median age of the overall cohort was 61 years (interquartile range [IQR], 54–66), with similar median ages observed in the double-hit (61 [54–65]) and single-hit (62 [53–68]) groups.

Female patients accounted for 45% of the double-hit group and 52% of the single-hit group. The cohort was predominantly White (65%), followed by African American (25%), with smaller proportions of Asian (3.9%), Hispanic (1.9). At diagnosis, most patients had preserved performance status, with 112 patients (73%) having an ECOG performance status of 1 and 24 (16%) having an ECOG of 0. According to the Revised International Staging System (R-ISS), 8 (5.2%) had stage I disease, 87 patients (56%) had stage II disease, and 53 (34%) had stage III disease.

With respect to disease subtype, IgG kappa was the most prevalent myeloma isotype (36%), followed by IgG lambda (21%). Among high-risk cytogenetic abnormalities, the gain of chromosome 1q was the most frequently observed abnormality, present in 89 patients (58%), followed by t(4;14) in 59 (38%), del(17p) in 26 (17%), and t(14;16) in 23 (15%). Gain of 1q and t(4;14) were more frequently observed in the double-hit group, whereas del(17p) was more common in the single-hit group (Table 1). Baseline extramedullary disease (EMD) characteristics are summarized in Table 2. EMD was present in a minority of patients, with paraskeletal involvement observed in 7.1% and extraosseous disease in 2.6% of the overall cohort. The distribution of EMD was comparable between the double-hit and single-hit groups.

**Table 1:** Baseline Demographic and Clinical Characteristics of the Study Population.

Characteristic	Overall N = 154 ^1^	Double-Hit N = 91 ^1^	Single-Hit N = 63 ^1^	*p*-Value ^2^
Age (IQR)	61 (54, 66)	61 (54, 65)	62 (53, 68)	0.4
Unknown	1	1	0	
Gender				0.4
F	74 (48%)	41 (45%)	33 (52%)	
M	80 (52%)	50 (55%)	30 (48%)	
Race				0.2
AA	39 (25%)	27 (30%)	12 (19%)	
Asian	6 (3.9%)	1 (1.1%)	5 (7.9%)	
Hispanic	3 (1.9%)	2 (2.2%)	1 (1.6%)	
Native American	1 (0.6%)	1 (1.1%)	0 (0%)	
Other	5 (3.2%)	3 (3.3%)	2 (3.2%)	
W	100 (65%)	57 (63%)	43 (68%)	
PS				0.4
0	24 (16%)	16 (18%)	8 (13%)	
1	112 (73%)	67 (74%)	45 (71%)	
2	17 (11%)	8 (8.8%)	9 (14%)	
3	1 (0.6%)	0 (0%)	1 (1.6%)	
Stage R-ISS				0.9
1	8 (5.4%)	4 (4.5%)	4 (6.8%)	
2	87 (59%)	53 (60%)	34 (58%)	
3	53 (36%)	32 (36%)	21 (36%)	
Unknown	6	2	4	
High Risk Cytogenetics				
t(4:14)	59 (38%)	37 (41%)	22 (35%)	
Del(17p)	26 (17%)	10 (11%)	16 (25%)	
t(14;16)	23 (15%)	14 (15%)	9 (14%)	
1q gain	89 (58%)	57 (63%)	32 (51%)	
Type of Myeloma				0.3
Free Kappa	15 (9.7%)	8 (8.8%)	7 (11%)	
Free Lambda	7 (4.5%)	5 (5.5%)	2 (3.2%)	
IgA Kappa	29 (19%)	15 (16%)	14 (22%)	
IgA Lambda	13 (8.4%)	9 (9.9%)	4 (6.3%)	
IgG Kappa	55 (36%)	37 (41%)	18 (29%)	
IgG Lambda	33 (21%)	17 (19%)	16 (25%)	
Non-sec	2 (1.3%)	0 (0%)	2 (3.2%)	

^1^ Median (Q1, Q3); n (%); ^2^ Wilcoxon rank sum test; Pearson’s Chi-squared test; Fisher’s exact test. Abbreviations: AA, African American; Del(17p), deletion of chromosome 17p; F, female; IgA, immunoglobulin A; IgG, immunoglobulin G; M, male; Non-sec, non-secretory; PS, performance status; R-ISS, Revised International Staging System; W, White; IQR, interquartile range.

**Table 2:** Baseline distribution of extramedullary disease in transplant-eligible patients with high-risk multiple myeloma.

Characteristic	Overall N = 154 ^1^	Double-Hit N = 91 ^1^	Single-Hit N = 63 ^1^
EMD Status			
Para-skeletal (Bone-related)	11 (7.1%)	6 (6.6%)	5 (7.9%)
Plasmacytoma	7 (4.5%)	2 (2.2%)	5 (7.9%)
Spine/Sacrum/Sacral Soft Tissue	4 (2.6%)	4 (4.4%)	0 (0.0%)
Extraosseous (Soft Tissue/Organ)	4 (2.6%)	2 (2.2%)	2 (3.2%)
CNS/orbital	1 (0.6%)	0 (0.0%)	1 (1.6%)
Lymph nodes	1 (0.6%)	0 (0.0%)	1 (1.6%)
Muscle/Hepatic	1 (0.6%)	1 (1.1%)	0 (0.0%)
Pleural	1 (0.6%)	1 (1.1%)	0 (0.0%)
No EMD (N/A)	139 (90.3%)	83 (91.2%)	56 (88.9%)

^1^ n (%). Abbreviations: EMD, extramedullary disease; CNS, central nervous system; N/A, not applicable.

### 3.2. Response to Induction Therapy

Response outcomes following induction therapy are summarized in Table 3. After first-line induction, the overall response rate (≥PR) in the entire cohort was 88%, with comparable response rates observed in patients with double-hit (87%) and single-hit (89%) HRMM, without a statistically significant difference between groups (*p* = 0.7). The distribution of best responses after first induction, including sCR, CR, VGPR, PR, stable disease, and PD, was also similar between cytogenetic subgroups, with no significant difference in depth of response (*p* = 0.3).

A subset of patients underwent a second induction regimen. Among these patients, the overall response rate was 65% in the overall cohort and was identical in both the double-hit (65%) and single-hit (65%) groups, with no significant difference in response achievement (*p* > 0.9). Similarly, the distribution of best responses following the second induction did not differ significantly between cytogenetic subgroups (*p* = 0.8). As expected in this real-world cohort, response assessment after second induction was not evaluable in a substantial proportion of patients, because some patients received only 1–2 cycles of second-line induction before proceeding to the next treatment step. Frontline induction regimens are summarized in Table 4. The majority of patients received triplet-based therapy, most commonly bortezomib, lenalidomide, and dexamethasone (60% overall; 64% in the double-hit group and 54% in the single-hit group). Cyclophosphamide, bortezomib, and dexamethasone was the second most frequently used regimen (14% overall), followed by smaller proportions receiving carfilzomib-based combinations (4.5%). Quadruplet therapy with daratumumab, bortezomib, lenalidomide, and dexamethasone was used in a minority of patients (8.4%). Doublet regimens and intensive chemotherapy-based approaches were infrequently utilized. Overall, induction patterns were consistent with contemporary practice, with predominant use of proteasome inhibitors and immunomodulatory drug-based combinations across both cytogenetic subgroups.

**Table 3:** Overall and Best Response Rates Following Induction Therapy.

	Overall (N = 154) ^1^	Double-Hit (N = 91) ^1^	Single-Hit (N = 63) ^1^	*p*-Value ^2^
ORR After First Induction				0.7
ORR achieved (≥PR)	135 (88%)	79 (87%)	56 (89%)	
No response (<PR)	19 (12%)	12 (13%)	7 (11%)	
Best Response After First Induction *				0.3
sCR	8 (5.5%)	7 (8.3%)	1 (1.6%)	
CR	29 (20%)	15 (18%)	14 (23%)	
VGPR	56 (39%)	32 (38%)	24 (39%)	
PR	40 (28%)	24 (29%)	16 (26%)	
SD	2 (1.4%)	0 (0%)	2 (3.3%)	
PD	10 (6.9%)	6 (7.1%)	4 (6.6%)	
Not evaluable	9	7	2	
ORR After Second Induction **				>0.9
ORR achieved (≥PR)	31 (65%)	20 (65%)	11 (65%)	
No response (<PR)	17 (35%)	11 (35%)	6 (35%)	
Not evaluable	106	60	46	
Best Response After Second Induction				0.8
sCR	1 (3.0%)	1 (4.5%)	0 (0%)	
CR	8 (24%)	6 (27%)	2 (18%)	
VGPR	20 (61%)	13 (59%)	7 (64%)	
PR	4 (12%)	2 (9.1%)	2 (18%)	
Not evaluable	121	69	52	

^1^ n (%); ^2^ Pearson’s Chi-squared test; Fisher’s exact test. Abbreviations: ORR, overall response rate; sCR, stringent complete response; CR, complete response; VGPR, very good partial response; PR, partial response; SD, stable disease; PD, progressive disease. * ORR defined as ≥PR per IMWG criteria. ** ORR post-second induction is shown as: ORR achieved (≥PR)—n (%), No response (<PR)—n (%), Not evaluable—n (%).

**Table 4:** Distribution of frontline induction regimens in transplant-eligible patients with high-risk multiple myeloma.

Characteristic	Overall N = 154 ^1^	Double-Hit N = 91 ^1^	Single-Hit N = 63 ^1^
Induction Therapy			
Doublet			
Rd	2 (1.3%)	0 (0%)	2 (3.2%)
Vd	2 (1.3%)	2 (2.2%)	0 (0.0%)
TD	2 (1.3%)	0 (0%)	2 (3.2%)
Triplet			
VRd/RVd	92 (60%)	58 (64%)	34 (54%)
CyBorD	22 (14%)	12 (13.2%)	10 (15.9%)
KRd	7 (4.5%)	5 (5.5%)	2 (3.2%)
KCd	2 (1.3%)	0 (0.0%)	2 (3.2%)
VTd	2 (1.3%)	2 (2.2%)	0 (0.0%)
DRd	2 (1.3%)	1 (1.1%)	1 (1.6%)
DVd	1 (0.6%)	1 (1.1%)	0 (0.0%)
Quadruplet			
Dara-VRd	13 (8.4%)	6 (6.6%)	7 (11.1%)
Intensive			
D-PACE	1 (0.6%)	1 (1.1%)	0 (0.0%)
VDPACE	2 (1.3%)	2 (2.2%)	0 (0.0%)
VTDPACE	2 (1.3%)	0 (0.0%)	2 (3.2%)
Linvoseltamab	1 (0.6%)	0 (0.0%)	1 (1.6%)
N/A	1 (0.6%)	1 (1.1%)	0 (0.0%)

^1^ n (%). Abbreviations: VRd, bortezomib/lenalidomide/dexamethasone; RVd, lenalidomide/bortezomib/dexamethasone; CyBorD, cyclophosphamide/bortezomib/dexamethasone; KRd, carfilzomib/lenalidomide/dexamethasone; KCd, carfilzomib/cyclophosphamide/dexamethasone; VTd, bortezomib/thalidomide/dexamethasone; DRd, daratumumab/lenalidomide/dexamethasone; DVd, daratumumab/bortezomib/dexamethasone; Dara-VRd, daratumumab/bortezomib/lenalidomide/dexamethasone; Rd, lenalidomide/dexamethasone; Vd, bortezomib/dexamethasone; TD, thalidomide/dexamethasone; D-PACE, dexamethasone/cisplatin/doxorubicin/cyclophosphamide/etoposide; VDPACE, bortezomib/dexamethasone/cisplatin/doxorubicin/cyclophosphamide/etoposide; VTDPACE, bortezomib/thalidomide/dexamethasone/cisplatin/doxorubicin/cyclophosphamide/etoposide.

### 3.3. Response Following Autologous Stem Cell Transplantation

Post-transplant response outcomes are summarized in Table 5. Following ASCT, the overall response rate (ORR; ≥PR) for the entire cohort was 88%. Patients with single-hit HRMM demonstrated a significantly higher ORR compared with those with double-hit disease (97% vs. 82%, *p* = 0.006).

Despite this difference in overall response achievement, the depth of response following ASCT was similar between cytogenetic subgroups. Rates of sCR were 21% in the double-hit group and 13% in the single-hit group, while CR rates were 32% and 40%, respectively. VGPR was observed in 33% of double-hit and 37% of single-hit patients, and PR occurred in 3.5% and 6.5%, respectively. PD rates remained low in both groups 11% (DHMM) vs. 3.2% (SHMM). Overall, no statistically significant difference was observed in the distribution of best response categories after ASCT (*p* = 0.2). Post-transplant response was unavailable for a small number of patients in both groups.

**Table 5:** Overall and Best Response Rates Following Autologous Stem Cell Transplantation.

Characteristic	Overall N = 154 ^1^	Double-Hit N = 91 ^1^	Single-Hit N = 63 ^1^	*p*-Value ^2^
Response Status After ASCT *				0.006
N	18 (12%)	16 (18%)	2 (3.2%)	
Y	136 (88%)	75 (82%)	61 (97%)	
Best Response After ASCT				0.2
CR	52 (35%)	27 (32%)	25 (40%)	
PD	11 (7.5%)	9 (11%)	2 (3.2%)	
PR	7 (4.8%)	3 (3.5%)	4 (6.5%)	
sCR	26 (18%)	18 (21%)	8 (13%)	
VGPR	51 (35%)	28 (33%)	23 (37%)	
Unknown	7	6	1	
ORR achieved (≥PR)	136 (88%)	75 (82%)	61 (97%)	
No response (<PR)	18 (12%)	16 (18%)	2 (3%)	

^1^ n (%); ^2^ Pearson’s Chi-squared test; Fisher’s exact test; * Responder status was defined as CR or PR per Lugano criteria at post-ASCT assessment; SD or PD were classified as non-response. Abbreviations: N, No; Y, Yes; ASCT, autologous stem cell transplantation; CR, complete response; ORR, overall response rate; PD, progressive disease; PR, partial response; sCR, stringent complete response; VGPR, very good partial response.

### 3.4. Treatment Exposure and Maintenance Therapy After ASCT

Post-transplant maintenance therapy patterns and response at 6 months are summarized in Table 6. Maintenance strategies were distributed similarly between cytogenetic subgroups. Overall, 44.8% of patients received IMiD-based maintenance, 23.4% received combined IMiD plus proteasome inhibitor (PI) therapy, 10.4% received PI-based maintenance alone, and 8.4% received anti-CD38-based maintenance. No statistically significant differences in maintenance class distribution were observed between patients with single-hit and double-hit high-risk HRMM. Treatment exposure and refractory status between single-hit and double-hit HRMM are summarized in Table 7.

At 6 months following initiation of maintenance therapy, the distribution of best responses was comparable between cytogenetic subgroups, with no statistically significant difference observed (*p* = 0.2). CR was achieved in 31% of the overall cohort, including 25% of double-hit and 39% of single-hit patients. Rates of stringent sCR were 19% in the double-hit group and 13% in the single-hit group, while VGPR occurred in 19% and 22%, respectively. PD at 6 months was observed in 21% of double-hit patients compared with 9.3% of single-hit patients. A substantial proportion of patients in both groups had either an undocumented depth of response despite response achievement or missing response assessments, reflecting real-world follow-up limitations.

**Table 6:** Post-Transplant Maintenance Therapy and Response at 6 Months.

Maintenance Class	Overall 154 (%) ^1^	Double Hit 91 (%) ^1^	Single Hit 63 (%) ^1^	*p*-Value ^2^
IMiD based	69 (44.8%)	42 (46.2%)	27 (42.9%)	
PI-based	16 (10.4%)	7 (7.7%)	9 (14.3%)	
IMiD + PI	36 (23.4%)	23 (25.3%)	13 (20.6%)	
Anti-CD38-based	13 (8.4%)	7 (7.7%)	6 (9.5%)	
No/unknown maintenance	20 (13.0%)	12 (13.2%)	8 (12.7%)	
Best Response After 6 Months of Maintenance Therapy				0.2
CR	40 (31%)	19 (25%)	21 (39%)	
PD	21 (16%)	16 (21%)	5 (9.3%)	
PR	1 (0.8%)	0 (0%)	1 (1.9%)	
sCR	22 (17%)	15 (19%)	7 (13%)	
SD	2 (1.5%)	2 (2.6%)	0 (0%)	
VGPR	27 (21%)	15 (19%)	12 (22%)	
Unknown or Response achieved, depth not specified	41 (26.6%)	24 (26.4%)	17 (27%)	

^1^, n (%), ^2^, Pearson’s Chi-squared test; Fisher’s exact test; Abbreviations: Anti-CD38, anti-cluster of differentiation 38 monoclonal antibody; CR, complete response; IMiD, immunomodulatory drug; PD, progressive disease; PI, proteasome inhibitor; PR, partial response; sCR, stringent complete response; SD, stable disease; VGPR, very good partial response.

**Table 7:** Treatment Exposure and Refractory Status.

Characteristic	Overall N = 154 ^1^	Double-Hit N = 91 ^1^	Single-Hit N = 63 ^1^	*p*-Value ^2^
Velcade exposure				0.6
N	12 (7.8%)	6 (6.6%)	6 (9.5%)	
Y	142 (92%)	85 (93%)	57 (90%)	
Velcade refractory				0.4
N	122 (79%)	70 (77%)	52 (83%)	
Y	32 (21%)	21 (23%)	11 (17%)	
Carfilzomib exposure				>0.9
N	119 (77%)	70 (77%)	49 (78%)	
Y	35 (23%)	21 (23%)	14 (22%)	
Carfilzomib refractory				0.8
N	133 (86%)	78 (86%)	55 (87%)	
Y	21 (14%)	13 (14%)	8 (13%)	
PI exposure				0.064
N	8 (5.2%)	2 (2.2%)	6 (9.5%)	
Y	146 (95%)	89 (98%)	57 (90%)	
PI refractory				0.4
N	119 (77%)	68 (75%)	51 (81%)	
Y	35 (23%)	23 (25%)	12 (19%)	
Len exposure				0.5
N	15 (9.7%)	10 (11%)	5 (7.9%)	
Y	139 (90%)	81 (89%)	58 (92%)	
Len refractory				0.7
N	122 (79%)	71 (78%)	51 (81%)	
Y	32 (21%)	20 (22%)	12 (19%)	
IMiD exposure				0.7
N	14 (9.1%)	9 (9.9%)	5 (7.9%)	
Y	140 (91%)	82 (90%)	58 (92%)	
IMiD refractory				0.7
N	120 (78%)	70 (77%)	50 (79%)	
Y	34 (22%)	21 (23%)	13 (21%)	
Dara exposure				0.064
N	108 (70%)	69 (76%)	39 (62%)	
Y	46 (30%)	22 (24%)	24 (38%)	
Dara refractory				0.6
N	125 (81%)	75 (82%)	50 (79%)	
Y	29 (19%)	16 (18%)	13 (21%)	
Triple RRMM				0.7
N	131 (86%)	76 (85%)	55 (87%)	
Y	21 (14%)	13 (15%)	8 (13%)	
Unknown	2	2	0	

^1^ n (%); ^2^ Fisher’s exact test; Pearson’s Chi-squared test; Abbreviations: Dara, daratumumab; IMiD, immunomodulatory drug; Len, lenalidomide; PI, proteasome inhibitor; RRMM, relapsed/refractory multiple myeloma.

### 3.5. Progression Free Survival and Overall Survival

**Figure 1:**
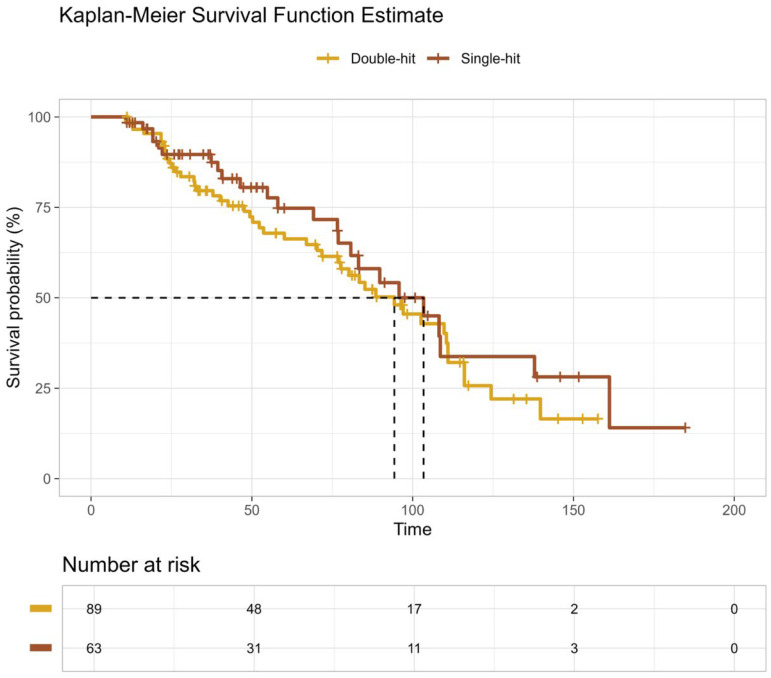
Overall Survival According to High-Risk Cytogenetic Burden. Kaplan–Meier estimates of overall survival (OS) following upfront autologous stem cell transplantation in patients with high-risk multiple myeloma stratified by cytogenetic burden. Patients with double-hit high-risk cytogenetics (gold line) demonstrated inferior OS compared with those with single-hit high-risk cytogenetics (brown line). Overall survival did not differ significantly between patients with double-hit and single-hit high-risk cytogenetics (log-rank *p* = 0.318).

**Figure 2:**
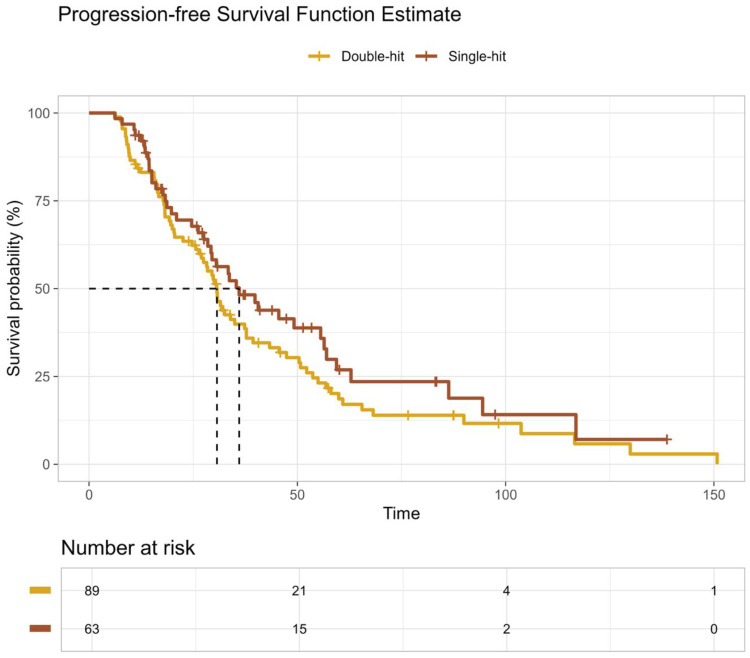
Progression-Free Survival According to High-Risk Cytogenetic Burden. Kaplan–Meier estimates of progression-free survival (PFS) following upfront autologous stem cell transplantation in patients with high-risk multiple myeloma stratified by cytogenetic burden. Patients with double-hit high-risk cytogenetics (gold line) experienced inferior PFS compared with those with single-hit high-risk cytogenetics (brown line). Progression-free survival did not differ significantly between patients with double-hit and single-hit high-risk cytogenetics (log-rank *p* = 0.213).

Overall survival (OS) and progression-free survival (PFS) outcomes following ASCT are summarized in Table 8 and illustrated in Figure 1 and Figure 2, respectively. With a median follow-up of 150 months for OS, a total of 70 deaths were observed among 152 patients. Median OS was 94 months (95% CI, 77–111) in the double-hit HRMM group and 103 months (95% CI, 81—not reached) in the single-hit group, with no statistically significant difference between groups by log-rank testing (*p* = 0.40; Figure 1; Table 8A). Restricted mean survival time (RMST) at 150 months was 87.4 months for double-hit patients and 96.6 months for single-hit patients, with no significant difference observed (*p* = 0.318).

For PFS, with a median follow-up of 125 months, 115 progression or death events were recorded. Median PFS was 31 months (95% CI, 27–38) in the double-hit group and 36 months (95% CI, 29–56) in the single-hit group. This difference was not statistically significant by log-rank testing (*p* = 0.20; Figure 2; Table 8B). RMST for PFS at 125 months was 41.3 months in the double-hit group and 49.6 months in the single-hit group, with no significant difference between groups (*p* = 0.213).

**Table 8:** Median Overall Survival (A) and Progression-Free Survival (B) After Autologous Stem Cell Transplantation.

(A)				
Characteristic	N	Event N	Median OS (in Months) *	*p*-Value ^1^
Hit type	152	70		0.4
Double hit			94 (77, 111)	
Single hit			103 (81, NR)	
(B)				
Characteristic	N	Event N	Median PFS (in months) **	*p*-value ^1^
Hit type	152	115		0.2
Double hit			31 (27, 38)	
Single hit			36 (29, 56)	

^1^ Log-rank test; * At follow-up of 150 months, the restricted mean survival time is 87.394 months for the Double-hit group and 96.626 months for the Single-hit group, and the *p*-value for the difference is 0.318. ** At follow-up of 125 months, the restricted mean progression-free survival time is 41.305 months for the Double-hit group and 49.593 months for the Single-hit group, and the *p*-value for the difference is 0.213. No statistically significant differences were observed (log-rank *p* > 0.05). Abbreviations: OS, overall survival; PFS, progression-free survival; NR, Not Reached; Event N, indicates the number of observed events (death for OS; progression or death for PFS).

## 4. Discussion

In this real-world multicenter analysis of 154 newly diagnosed patients with HRMM undergoing frontline ASCT, patients with double-hit cytogenetic abnormalities demonstrated progression-free survival (PFS) and overall survival (OS) comparable to those with single-hit abnormalities. Although patients with double-hit cytogenetic abnormalities achieved numerically lower complete response (CR) rates following ASCT and maintenance therapy, these differences were not statistically significant. Collectively, these findings contrast with earlier reports of markedly inferior outcomes in double-hit disease and suggest that advances in induction regimens, maintenance strategies, and salvage therapies may attenuate the adverse prognostic impact of high-risk cytogenetic abnormalities [1,5,9]. Importantly, this study was designed to evaluate intra-high-risk heterogeneity by comparing outcomes between single-hit and double-hit cytogenetic subgroups within a contemporary, transplant-eligible real-world cohort. Accordingly, a standard-risk comparator group was not included, and the findings should be interpreted within the context of risk stratification within high-risk disease rather than across cytogenetic risk categories.

Earlier studies consistently demonstrated markedly inferior outcomes among patients harboring two or more high-risk cytogenetic abnormalities, particularly when involving del(17p), t(4;14), or 1q gain/amplification. The 2016 CIBMTR analysis by Scott et al. demonstrated poor survival among patients with ≥2 high-risk abnormalities, with 3-year PFS and OS of 27% and 67%, respectively; however, this subgroup included only 27 patients, limiting statistical power and generalizability [7,10]. Similarly, genomic analyses defining “double-hit” myeloma emphasized its aggressive biology and inferior prognosis in earlier treatment eras [7,11].

Prospective trial data have also demonstrated inferior outcomes in patients with multiple high-risk features. The FORTE trial reported 4-year PFS and OS of 39% and 63% in patients with ≥2 high-risk abnormalities, underscoring the adverse prognostic impact of cytogenetic complexity despite modern induction strategies [3,12]. In contrast, the median PFS (31 months) and OS (94 months) observed in our double-hit cohort are substantially more favorable, suggesting that real-world application of contemporary treatment paradigms may mitigate (but not eliminate) cytogenetic risk. While outcomes in our cohort appear favorable compared with historical double-hit populations, they remain generally inferior to those typically reported in standard-risk multiple myeloma in prior studies, supporting the continued adverse prognostic significance of high-risk cytogenetic disease.

Real-world studies have highlighted pronounced heterogeneity within the double-hit category. Marcoux et al. reported extremely poor outcomes in patients with concurrent del(17p) and t(4;14), with median PFS and OS of 11.5 and 22.4 months, respectively, emphasizing that not all double-hit combinations carry equivalent risk [13,14]. Conversely, Pasvolsky et al. reported more favorable outcomes in a cohort of 79 patients with ≥2 high-risk abnormalities treated with ASCT, with median PFS of 22.9 months and OS of 71.5 months, findings more consistent with our results [15]. Collectively, these data suggest that improvements in therapy, patient selection, and supportive care have progressively improved outcomes compared with historical cohorts.

Several factors likely contributed to the favorable outcomes observed in our cohort. First, proteasome inhibitor-based induction therapy was used in the vast majority of patients, with VRd administered in over half. Proteasome inhibitors have been shown to partially overcome adverse cytogenetic risk, particularly in patients with t(4;14) and gain(1q) abnormalities [1,3,9]. High overall response rates following induction and ASCT in both groups are consistent with prior observations that depth of response correlates strongly with improved survival in high-risk disease [3,12].

Second, post-transplant maintenance therapy was widely utilized, with most patients receiving lenalidomide-based or proteasome inhibitor-containing regimens. Multiple randomized trials and real-world analyses have demonstrated that maintenance therapy prolongs PFS and, in some studies, OS, even among high-risk patients [9,12]. Extended genetic profiling suggests that maintenance therapy may differentially benefit subsets of high-risk patients, potentially contributing to the narrowing of outcome differences between single- and double-hit disease [12].

Third, our study period spans the era of highly effective salvage therapies, including anti-CD38 monoclonal antibodies, next-generation proteasome inhibitors, immunomodulatory agents, and, more recently, T-cell-directed immunotherapies such as bispecific antibodies and CAR T-cell therapies. The availability of multiple effective post-relapse treatment options likely contributed to the prolonged OS observed despite relatively modest PFS durations, consistent with prior real-world and translational studies [16,17,18].

Fourth, high-dose melphalan conditioning followed by ASCT remains a cornerstone of therapy for transplant-eligible high-risk patients. Despite the introduction of numerous novel agents, ASCT continues to provide deep cytoreduction and remains associated with durable disease control, even in double-hit and ultra-high-risk populations, when integrated with modern induction and maintenance approaches [1,6,19].

The lack of statistically significant differences in PFS and OS between double-hit and single-hit patients warrants careful interpretation. While the observed trend toward inferior PFS in double-hit patients aligns with established prognostic paradigms, the modest absolute difference may reflect genuine improvements in outcomes for double-hit disease in the modern era [5,20].

Additionally, emerging evidence suggests that response depth, clonal architecture, and genomic complexity influence outcomes beyond individual cytogenetic lesions [10,21]. The high rates of ≥VGPR and sustained maintenance therapy in our cohort may have partially offset the adverse impact of multiple high-risk abnormalities.

Importantly, prognostic heterogeneity within the double-hit category is increasingly recognized. Specific combinations such as del(17p) with t(4;14), del(17p) with gain(1q), or MYC rearrangements have been associated with particularly aggressive disease biology and inferior outcomes. In our cohort, the predominance of gain(1q)-based combinations may partially explain the relatively favorable outcomes observed compared with cohorts enriched for TP53-disrupted disease [11,13,22,23,24]. Additionally, depth and durability of response, adherence to maintenance therapy, and quality of supportive care may mitigate adverse cytogenetic risk. High rates of very good partial response or better and sustained maintenance therapy utilization in our cohort likely contributed to improved disease control [12,19].

These findings have several important clinical implications. First, the comparable survival outcomes observed support the continued use of frontline ASCT in transplant-eligible patients with double-hit HRMM, provided it is delivered within a framework of effective induction and sustained maintenance therapy. Second, the apparent benefit of maintenance therapy reinforces its role as standard of care for high-risk patients following ASCT, consistent with expert consensus recommendations [9]. Third, prolonged OS despite earlier relapse underscores the importance of access to effective salvage therapies, including novel immunotherapies, for high-risk patients. Ensuring equitable access to these treatments will be critical to maintaining and further improving survival outcomes [16,17,18]. Finally, the persistent trend toward inferior PFS in double-hit disease highlights the need for continued therapeutic innovation, including intensified induction regimens, antibody-based maintenance, MRD-adapted strategies, and early integration of novel immunotherapies [19,20].

This study has several limitations. Its retrospective design introduces potential selection and treatment biases, and restriction to transplant-eligible patients may preferentially include individuals with more favorable performance status and organ function, potentially underrepresenting those with higher disease burden. Additionally, heterogeneity in induction regimens, maintenance strategies, and treatment eras (2009–2024) may have influenced outcomes and represents an inherent limitation of this retrospective real-world analysis. Measurable residual disease (MRD) data were not uniformly available across centers or time periods, precluding assessment of depth of molecular response. This represents a key limitation, as MRD negativity is increasingly recognized as a strong, independent prognostic factor that may refine risk stratification beyond conventional cytogenetic classification, particularly in high-risk and double-hit disease. Additionally, the sample size limited detailed subgroup analyses of specific cytogenetic combinations. Furthermore, tandem transplantation was not uniformly utilized and was excluded from this analysis; therefore, we cannot exclude the possibility that patients with higher-risk cytogenetic features may have been preferentially selected for this approach, which could influence observed outcomes. Finally, the absence of a contemporaneous standard-risk comparator cohort limits the ability to contextualize outcomes relative to broader multiple myeloma populations.

Future studies should incorporate standardized MRD assessment to determine whether MRD negativity can overcome adverse cytogenetic risk in double-hit myeloma. Larger collaborative efforts are needed to refine risk stratification based on specific cytogenetic combinations and to evaluate treatment intensification strategies tailored to ultra-high-risk disease biology. Integrative translational studies combining genomic and clinical data may further elucidate mechanisms of resistance and identify actionable therapeutic targets.

## 5. Conclusions

In this contemporary real-world multicenter analysis, patients with double-hit HRMM undergoing frontline ASCT achieved PFS and OS comparable to those with single-hit disease. These findings contrast with earlier reports of markedly inferior outcomes and suggest that modern therapeutic strategies have substantially improved prognosis in HRMM. Nevertheless, the persistent trend toward inferior PFS highlights the need for continued innovation and refined risk-adapted treatment approaches for this challenging patient population.

## Data Availability

The data presented in this study are available on request from the corresponding author.
